# Template-Free Synthesis of High Dehydration Performance CHA Zeolite Membranes with Increased Si/Al Ratio Using SSZ-13 Seeds

**DOI:** 10.3390/membranes14040078

**Published:** 2024-03-27

**Authors:** Jing Du, Jilei Jiang, Zhigang Xue, Yajing Hu, Bo Liu, Rongfei Zhou, Weihong Xing

**Affiliations:** State Key Laboratory of Materials-Oriented Chemical Engineering, College of Chemical Engineering, Nanjing Tech University, Nanjing 210009, Chinaliu@njtech.edu.cn (B.L.)

**Keywords:** pervaporation, vapor permeation, zeolite membrane, template-free, Si-rich CHA zeolite

## Abstract

Pervaporation is an energy-efficient alternative to conventional distillation for water/alcohol separations. In this work, a novel CHA zeolite membrane with an increased Si/Al ratio was synthesized in the absence of organic templates for the first time. Nanosized high-silica zeolite (SSZ-13) seeds were used for the secondary growth of the membrane. The separation performance of membranes in different alcohol–aqueous mixtures was measured. The effects of water content in the feed and the temperature on the separation performance using pervaporation and vapor permeation were also studied. The best membrane showed a water/ethanol separation factor above 100,000 and a total flux of 1.2 kg/(m^2^ h) at 348 K in a 10 wt.% water–ethanol mixed solution. A membrane with high performance and an increased Si/Al ratio is promising for the application of alcohol dehydration.

## 1. Introduction

Pervaporation (PV) and vapor permeation (VP) are promising alternative technologies to distillation for the energy-saving separation of azeotropic and near-azeotropic mixtures such as water/ethanol (EtOH) and water/isopropyl alcohol (IPA) [1,2,3,4,5,6]. Zeolite membranes have become the most popular among polymer [7,8], zeolite [9,10,11,12,13], and silica [14,15] membranes because of their excellent separation performance and improved hydrothermal stability. Sodium A (NaA) zeolite membranes with LTA framework had been commercialized for the PV and VP dehydration of water/alcohol mixtures [16,17]. It is a popular rule that the resistance of a zeolite to water steam and acids increases, but hydrophilicity decreases as the Si/Al ratio in the zeolite increases [18,19,20,21]. NaA zeolites possess the lowest Si/Al ratio, close to 1, suggesting that NaA zeolite membranes could display high dehydration performance but limited stability under hydrothermal and acidic conditions. It was also proven via evidence in the literature that NaA zeolite membranes were not stable in a mixture with water higher than 30% at high temperatures, or a mixture with a few acetic acids [22,23]. FAU zeolite membranes with Si/Al ratios of 1–3 displayed high water fluxes but relatively low selectivities, because the size of their 12-member-ring pore of 0.74 nm × 0.74 nm was larger than the diameters of water and EtOH (or IPA). Similarly, mordenite and ZSM-5 membranes displayed relatively low water/alcohol separation factors because their pore sizes were larger than the kinetic diameters of water and alcohols, even if they displayed good resistance to acids, because Si/Al ratios in their frameworks (5–10) were higher than that in FAU and LTA zeolite frameworks. Zeolite T (an intergrown zeolite of erionite and offretite) membranes with Si/Al ratios of 3–4 were stable in the weak acids with a pH value larger than 3 [24,25].

The CHA zeolite framework has 3D and interconnected channels with a window size of 0.38 nm × 0.38 nm. The straight and 8-member-ring pores enable CHA zeolite membranes to display excellent water (0.28 nm) selectivity by size exclusion over methanol (0.38 nm), EtOH (0.44 nm), and IPA (0.58 nm) when it grows to a continuous and uniform membrane. Chabazite membranes with a Si/Al ratio of 2–3.5 were normally prepared using the template-free route. Gu et.al. [18,26,27] reported that they synthesized high-flux chabazite membranes with a Si/Al ratio of 2.8 from a clear solution. The membrane showed a water flux of 13.3 kg/(m^2^ h) and a separation factor of 6000 for the PV dehydration of 10 wt.% water/EtOH mixture at 348 K. Furthermore, a chabazite membrane with a Si/Al ratio of 3.2 displayed a total flux of 4.1 kg/(m^2^ h) and a separation factor of 39,500, as reported by Hasegawa et al. [28]. These results show that CHA zeolite membranes are suitable for the dehydration of alcohols.

Increasing the Si/Al ratio of CHA zeolite membranes in the absence of an organic template is very difficult [29]. In our previous study, a high-silica CHA zeolite membrane (known as SSZ-13) with a Si/Al ratio of ~12 prepared with an organic template displayed an excellent water/acetic acid selectivity of over 10,000 in the water/acetic mixture with a pH of less than 2 [30]. These results further confirm that the stability against acidic and hydrothermal corrosion has a singly positive correlation with the Si/Al ratio in the zeolite framework. Moreover, the results suggest that the water/organic selectivity of a zeolite membrane is related not only to the Si/Al ratio, but also to the density of defect pores. When the pore size of zeolite membranes is between water and organic molecules (CHA zeolites for example), a defect-free high-silica zeolite membrane could still display a high water/organic selectivity [31]. Therefore, it is reasonable to construct a CHA zeolite membrane with an increased Si/Al ratio but low defect density in order to increase its hydrothermal and acidic stabilities, while maintaining high water/organic selectivities.

In this current study, a novel CHA zeolite membrane with a Si/Al ratio above 5 has been prepared in the absence of an organic template with the assistance of high-silica CHA (SSZ-13) seeds for the first time. Different alkalis, cesium hydroxide and sodium hydroxide, were used to replace the normal alkalis of sodium hydroxide and potassium hydroxide. The separation performance of the current CHA zeolite membranes was measured in water/methanol, water/EtOH, and water/IPA mixtures as a function of temperature via PV and VP experiments. The water/ethanol separation factor of the typical CHA zeolite membrane is above 100,000, which is even higher than that of the normal low-silica CHA zeolite membranes. Our findings suggest that the current high-performance CHA zeolite membranes with an increased Si/Al ratio should display promising prospects in actual applications in the field of water/organic separation.

## 2. Materials and Methods

### 2.1. Synthesis Procedure for CHA Zeolite Membranes

CHA zeolite membranes were prepared using an organic template-free recipe with a molar composition of 11.0 SiO_2_: 2.0 Al (OH)_3_: 0.8 CsOH: 6.0 NaOH: 3.0 NaF: 600 H_2_O. The addition of cesium hydroxide, CsOH, was used to increase the mineralization ability of the gel, since CsOH has a strong alkalinity. For a typical synthesis, 3.9 g of aluminum hydroxide (99%, Wako, Osaka, Japan) was added in a solution containing 6.1 g of sodium hydroxide (96%, Sinopharm, Beijing, China), 6.0 g of cesium hydroxide (Sigma-Aldrich, St. Louis, MO, USA, 50 wt.% in water), and 40.0 g of deionized water (homemade). The solution was heated and stirred until all the aluminum source was fully dissolved. When cooling to ambient temperature, 3.2 g of sodium fluoride (99%, Sinopharm Chemical Reagent Co., Ltd. China), 41.0 g of colloidal silica (LUDOX HS-40, 40 wt.% aqueous suspension, Sigma-Aldrich), and 203 g of deionized water (homemade) were added to the aluminate solution one-by-one. An emulsion hydrogel was obtained by stirring the mixture for 24 h aging at room temperature.

Tubular α-alumina supports, which had an asymmetric structure with a 200 nm pore size (thickness of about 20 μm) and a 1.0 μm pore microporous alumina body, were provided by Jiangsu Membrane Park Co., Nanjing, China. CHA zeolite membranes were synthesized by secondary growth on the outer surface of a 10 cm long α-alumina support. High-silica SSZ-13 seeds, which possess the same CHA framework as the current CHA zeolite membranes, were used to induce the formation of CHA zeolite membranes with an increased Si/Al ratio in the framework. The outer surface of the tubular support was coated with commercial nanosized SSZ-13 seeds (Dalian Zhuoran Tech. Co., Dalian, China, Si/Al ratio of 14.2) by rub-coating. The supports were wetted with EtOH before rub-coating and the wetted support was rolled in the dry seed powders. Then, the fingers wiped all areas of the outer surface of the tube back and forth for about 1.0 min to ensure seeds were coated on all areas of the support. Approximately 300 g of gel was added into a Teflon-lined autoclave. And then, the secondary growth of a membrane was carried out for 24–72 h at 423 K. After the reaction, the autoclave was cooled to ambient temperatures with tap water and the membrane was removed from the autoclave and washed several times with deionized water until the solution becomes neutral. Subsequently, the membranes were dried.

### 2.2. PV and VP Experiments

**Figure 1:**
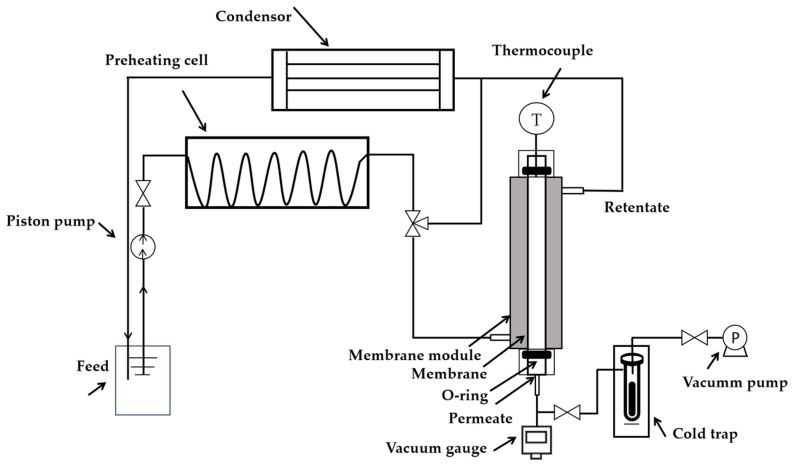
Schematic diagram of the PV experimental setup.

The as-synthesized membranes were measured for separation at 10/90 wt.% H_2_O/EtOH, 10/90 wt.% H_2_O/IPA, and 10/90 wt.% and 30/70 wt.% H_2_O/MeOH via PV and VP experiments. The PV experimental setup is shown in Figure 1. The liquid mixture was pumped to a preheating cell, to the set temperature, using a piston pump and then went through a membrane module, a cooling tank, and, finally, back to the feed tank. After reaching and stabilizing the desired temperature, the feed was directed to the membrane module via the three-way valve. The temperature of the feed was regulated, using thermocouples and temperature regulators, from room temperature to 150 °C. The retentate was condensed and returned to the feed tank. The permeate line was vacuumed to less than 100 Pa and the permeated vapor was iced in a cold trap filled with liquid nitrogen.

**Figure 2:**
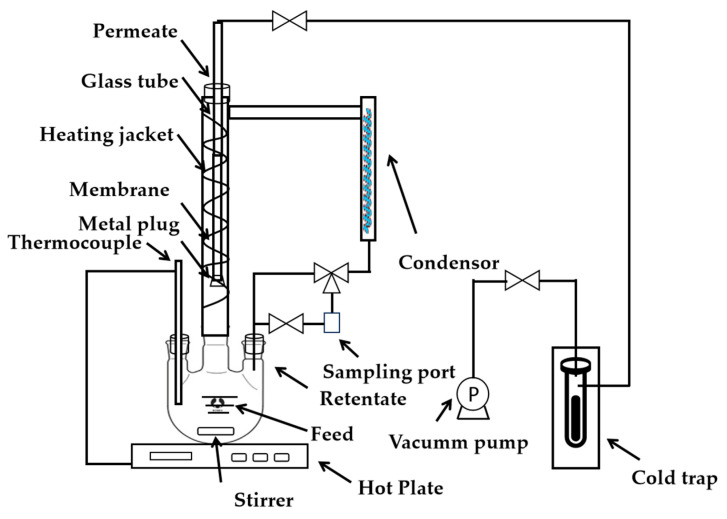
Schematic diagram of the VP experimental setup.

The VP experimental setup is shown in Figure 2. Before the VP experiments, the membrane was sealed. One end of the membrane was obstructed with a metal plug and the other end was connected to a glass tube using vacuum silicone grease and silicon rubber tubes. And then, the silicone tube was attached to a vacuum side. The liquid with a volume of about 500 mL in the three-necked flask was heated to the boiling point using a hot plate and then the vapor was further heated using a heating jacket to the set temperature (above boiling point), according to the gas–liquid equilibrium phase diagram of alcohol and water. Since the membrane is grown on the out surface of the tubular support, the membrane surface can easily contact the vapor directly. The vapor rose and then entered a water-cooling condenser to become liquid before flowing back to the flask via gravity. The condensed liquid mixture from the retentate could be collected through a three-way valve and was used to analyze the composition of the feed vapor mixture. The pressure in the three-mouthed flask is close to atmospheric pressure. A gas chromatograph (GC, GC-2014A, Shimadzu, Kyoto, Japan) was used to analyze the feed and permeate samples in the PV and VP tests.

The separation factor *α*, which is expressed by the mole fractions of the components in the feed and the permeate, respectively, is given in Equation (1). The permeation flux of each component or the total, *J*, which is expressed as the permeation amount in weight of each component or the total through the membrane per units of membrane area and time, is given in Equation (2).

(1) $$\alpha_{i/j}=\frac{yi/yj}{xi/xj}$$

(2) $$J=\frac{W}{\;\;A\cdot t}$$

where *y_i_*, *y_j_*, *x_i_*, and *x_j_* are weight fractions of component *i* (water) and component *j* (organic) in the permeate and feed, respectively. The samples of the permeate and feed were analyzed using the gas chromatograph (GC, GC-2014A, Shimadzu). *A*, *w*, and *t* are the membrane area (m^2^), the weight of permeating mass of each component or the total (kg), and the test time (h), respectively. The mass weight of the substance in the permeate side is obtained by weighing them on a balance. The more substances collected through the membrane on the permeate side per hour, the greater the flux of the membrane. The higher the flux and selectivity, the better the separation performance of the membrane.

### 2.3. Characterization

The morphologies and Si/Al ratios of zeolite membranes were characterized using Field Emission Scanning Electron Microscopy (FE-SEM, S-4800, Hitachi, Tokyo, Japan) with Cu-*Kα* radiation and an Energy Dispersive x-ray Detector (EDX, EMAX x-act, Horiba, Kyoto, Japan). The crystal phases and crystallinities were identified using X-ray diffraction (XRD) (Mini Flex 600, Rigaku, Tokyo, Japan).

## 3. Results and Discussion

### 3.1. Membrane Preparation

**Figure 3:**
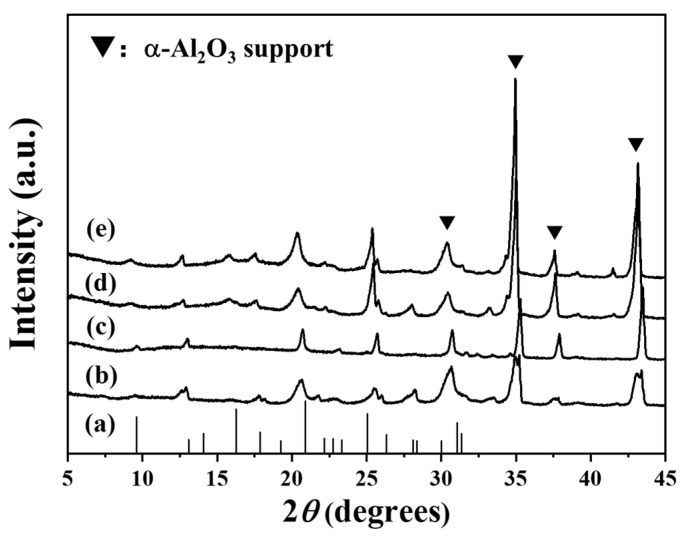
XRD patterns of (a) standard CHA zeolite and CHA zeolite membranes synthesized for (b) 24 h, (c) 36 h, (d) 48 h, and (e) 72 h.

To investigate the effects of reaction time on the growth of CHA zeolite membranes, membrane samples were comparably prepared by secondary growth at 423 K for 24–72 h. The XRD results in Figure 3 show that CHA zeolite membranes can be obtained for 24–72 h. It was well known that the crystallinity of zeolite always increased with the increase in synthesis time, until equilibrium is reached. For zeolite membranes, membrane thickness always increased with the extension of synthesis time before the dissolution was dominated. The characteristic peaks of the CHA zeolite increase with increasing synthesis time, indicating that crystallinity and the thickness of the membrane increase with the extension of synthesis time.

**Figure 4:**
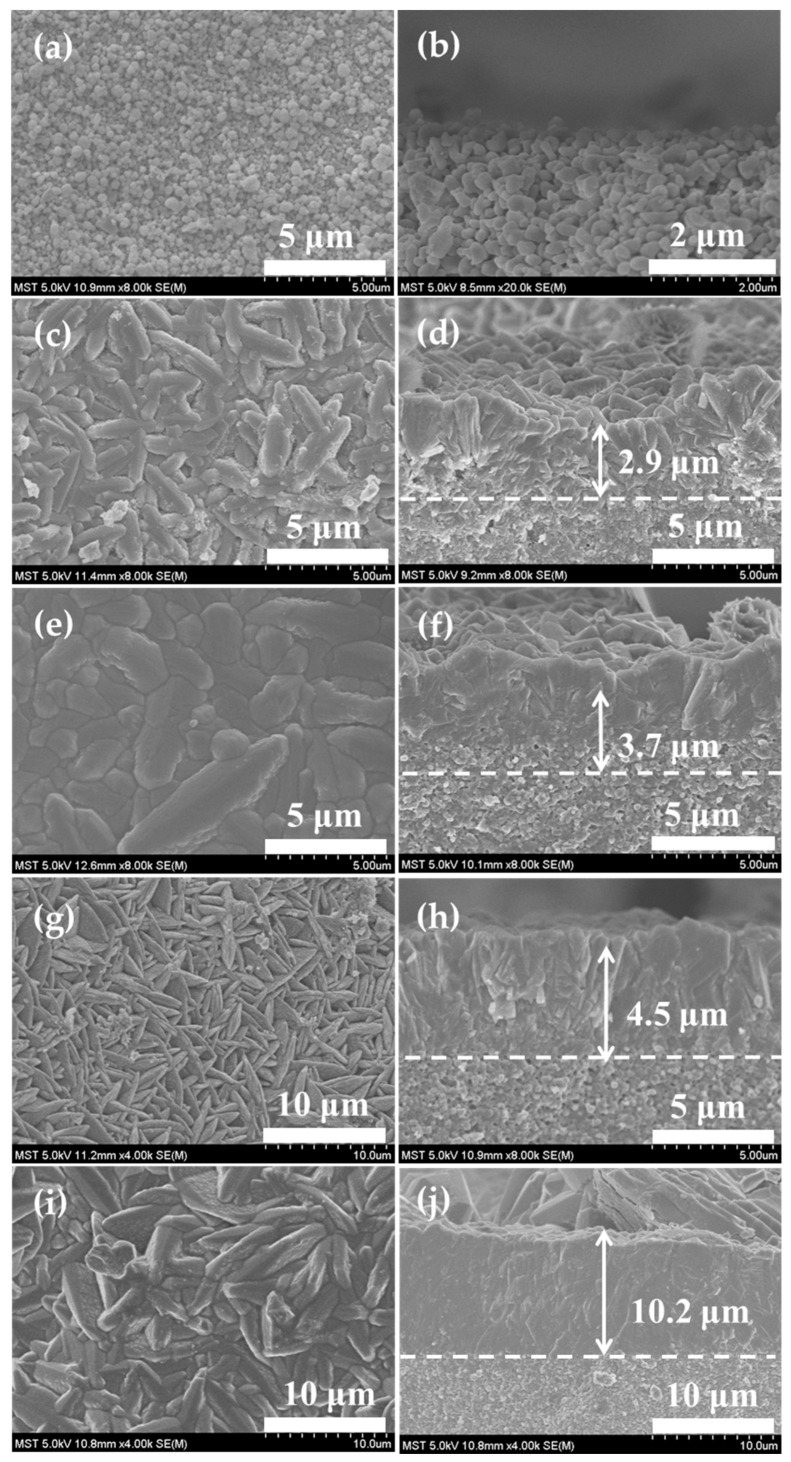
SEM images for the surface and cross section of (**a**,**b**) seeded support and CHA zeolite membranes at 423 K for (**c**,**d**) 24 h, (**e**,**f**) 36 h, (**g**,**h**) 48 h, and (**i**,**j**) 72 h, respectively.

Figure 4 displays SEM images of CHA zeolite membranes fabricated for various reaction times. A lot of walnut-shaped crystals were intergrown on the outer surface of the support. The walnut shape of the crystals in these membranes is the typical one, as has been reported in the literature [18,20]. However, the inter-crystalline pinhole defects were evident in the membrane layer, indicating that this membrane was discontinuous after only 24 h of synthesis (Figure 4d). The intergrowth behavior was improved and the membrane thickness increased from 2.9 to 10.2 µm, as reaction time was prolonged from 24 to 72 h (Figure 4c–j). A continuous and dense membrane with a thickness of 4.5 µm was formed after 48 h of synthesis (Figure 4g,h). Notably, the presence of a conspicuous seed composite layer close to the support layer (Figure 4d,f,h) demonstrated that the membrane layer was epitaxially grown upon the seeded layer. CHA zeolite membranes were formed by epitaxial growth of homogeneous and heterogeneous seed crystals [32,33,34]. These results indicated that the different values of the cross section came from the thickness increase, along with synthesis time in the time range. The intergrowth behavior was improved over time. Typically, alkali metal cations play a crucial role as mineralizers or inorganic structure directing agents during zeolite crystallization [32]. The utilization of cesium and sodium mixed cations could be a crucial issue to the crystallization of zeolite with CHA topology [35,36]. The use of mixed cations may promote the efficient nucleation of amorphous aluminosilicate gel, and the nuclei are subsequently deposited on the seed layer to facilitate the membrane growth. Ultimately, this process could lead to the formation of a continuous and dense membrane layer via the epitaxial growth of the seed layer.

**Figure 5:**
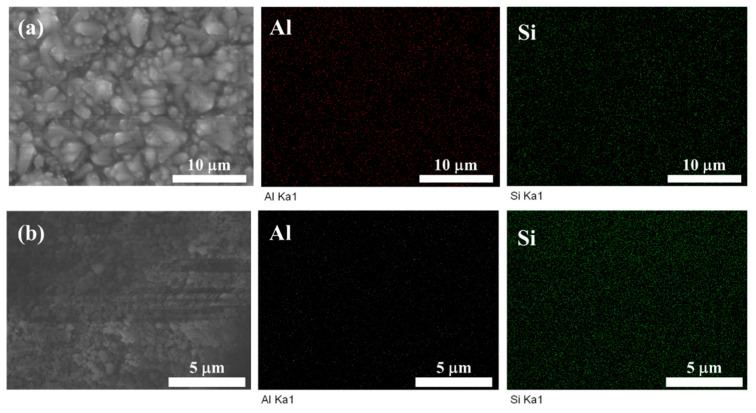
SEM images and corresponding EDX mappings of (**a**) surface of M3 and (**b**) SSZ-13 seeds.

EDX analysis was utilized to detect the Si/Al ratio of the CHA zeolite membrane. The Si/Al ratio of the membrane was 5.3, as shown in Figure 5 and Table 1. The synthesized CHA zeolite membrane had a higher Si/Al ratio than other CHA zeolite membranes prepared in the absence of an organic template (Si/Al < 3.5) [26,28]. In this study, the epitaxial growth of high-silica SSZ-13 seeds (Si/Al = 14.2) induced the formation of CHA zeolite membranes and led to the formation of membrane layers with an increased Si/Al ratio (5.3). This is consistent with previous findings that the utilization of zeolite seeds with different Si/Al ratios may lead to the formation of membrane layers with different Si/Al ratios via epitaxial growth [37].

### 3.2. PV and VP Performance

Table 2 shows the PV performance of these membranes prepared for different synthesis times in a 10/90 wt.% H_2_O/EtOH mixture. It was found that the flux of CHA zeolite membranes decreased over the reaction time, due to the increasing thickness of the membrane. However, H_2_O/EtOH selectivity had a maximum when the synthesis time was 48 h. The separation factor decreased when synthesis time increased from 48 to 72 h. It is mainly attributed to the overgrowth of membrane crystals. The particle size of the membrane crystals was as large as 10 μm (Figure 4i) and the overgrowth of the membrane crystals tended to form large inter-crystalline boundary defects [38]. Therefore, 48 h was the optimal synthesis time for our case. The best membrane, M5, showed a water/EtOH separation factor larger than 100,000, together with a flux of 1.1 kg/(m^2^ h) at 348 K in a 10/90 wt.% H_2_O/EtOH mixture. Four membranes were prepared under optimized conditions. All the membranes displayed excellent separation performances, with fluxes of 1.1–1.4 kg/(m^2^ h) and water/EtOH separation factors higher than 16,000. It suggests that the template-free synthesis of high-quality CHA zeolite membranes with enhanced Si/Al ratios is reproducible.

**Figure 6:**
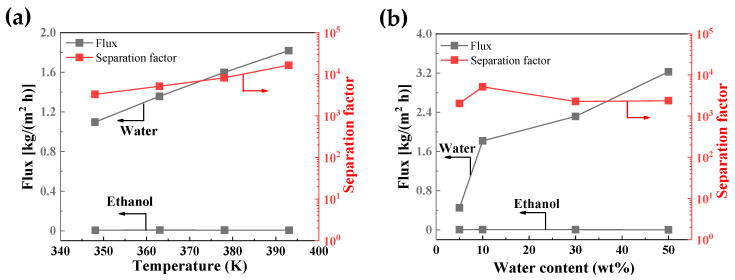
Water and ethanol fluxes and separation factors of membrane M2 as functions of (**a**) temperature and (**b**) water content in feed.

Figure 6 presents the effects of temperature and water content on the flux and separation factor of the membranes. As the test temperature rose from 348 (PV state) to 393 K (VP state), the flux of the membrane increased from 1.1 to 1.8 kg/(m^2^ h). It is interesting that the separation factor increased with temperature. The temperature dependence of the permeation property through zeolite membranes can be described in accordance with the Arrhenius-type equation [39]. The activated diffusion increased the permeation flux as feed vapor temperature increased. The water permeation flux through hydrophilic zeolite membranes was attributed to the combinatory effects of adsorption and diffusion [40]. According to the experimental results (Figure 6), the water flux increased along with temperature. It indicated that the permeation was dominated by activation diffusion through zeolite pores within the investigated temperature range. The ethanol flux also increased with increasing temperature, but the upward trend of ethanol was flatter than that of water. It could be attributed to the fact that the interaction of water–EtOH molecules reduces with temperature, resulting in an increasing diffusivity of water and increasing inhibition ability of water against the diffusion of EtOH through the membrane. Therefore, the water/EtOH separation factor increased. It agreed with the results for NaA membranes, as has been reported in the literature [41]. This result showed that CHA zeolite membranes could achieve water–alcohol separation over a wide range of temperatures in either pervaporation or vapor permeation, as shown in Figure 6a. The flux increased with water content in the feed, as shown in Figure 6b. A reason for this could be that the partial water pressure near the membrane surface increases with increasing water content in the bulk feed. The separation factor changed less with feed water content. Please note that the membrane still displayed a high separation factor even though the feed contained only 5 wt.% water.

Table 3 displays the VP performance of membrane M2 in three binary H_2_O/MeOH, H_2_O/EtOH, and H_2_O/IPA mixtures. The permeation property of zeolite membranes is related to the interaction forces between the guest molecules and the zeolite surface, as well as the forces between the guest molecules [42]. For water/alcohol mixtures, the small-size and high-polarity water molecules were preferentially adsorbed on the surface of the hydrophilic CHA zeolite, which may block the entrance of the alcohols in zeolite pores by the mechanisms of molecular sieving and preferential adsorption. The pore size of CHA (0.38 nm) is between water molecules (kinetic diameter of 0.27 nm) and alcohol molecules (methanol: 0.38 nm, ethanol: 0.44 nm, and isopropanol: 0.49 nm), indicating that the mechanism of molecular sieving benefits the water-selective separation over the alcohols. On the other hand, CHA zeolite membranes with a Si/Al ratio of 5.3 are still hydrophilic in nature. The water molecules could be preferentially adsorbed on the surface of CHA pores and pass quickly through the pore channels. The water molecules that occupy the pores will impede the permeation of the weakly adsorbed alcohol molecules, resulting in an excellent dehydration performance of the membrane. Moreover, the interaction force between isopropanol molecules and CHA pores is rather weaker than that between ethanol and CHA pores. The permeation of methanol inhibits the permeation of water molecules, which results in a relatively low flux and separation factor in the water/methanol mixture. Water molecules tend to occupy CHA pores more readily in an H_2_O/IPA system, thereby inhibiting the permeation of isopropanol to a greater extent. As a result, the flux rose from 0.6 to 2.5 kg/(m^2^ h) in the following order: H_2_O/MeOH < H_2_O/EtOH < H_2_O/IPA, which was consistent with the results in some cases using NaA and CHA zeolite membranes [40,43].

**Figure 7:**
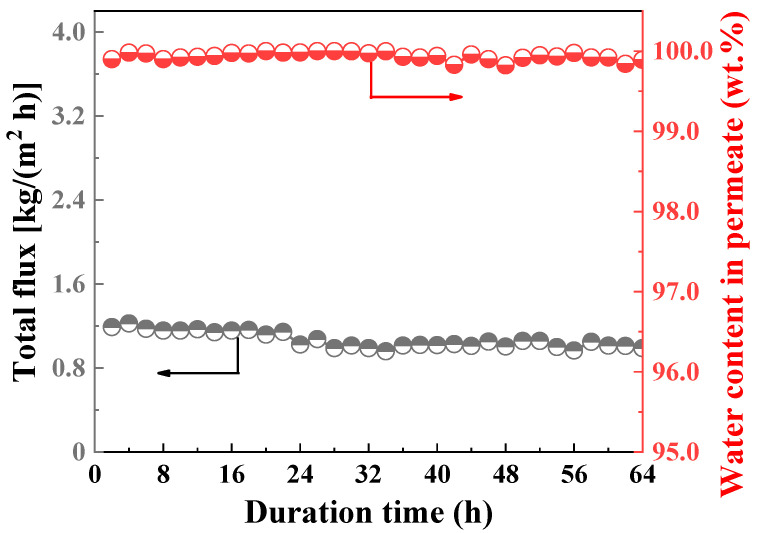
Evaluation of long-term stability of CHA zeolite membrane (M3) in a 10/90 wt.% H_2_O/EtOH mixture at different temperatures for 64 h.

Figure 7 reveals the long-term stability test of CHA zeolite membrane (M3) at 348 K in a 10/90 wt.% H_2_O/EtOH mixture for 64 h. The permeated samples were obtained every 2 h. According to the experimental results shown in Figure 7, the membranes maintained stable flux and separation factors under the test conditions. The flux decreased in the initial stage, probably because it took time to reach adsorption–desorption equilibrium. Then, the flux stabilized around 1–1.1 kg/(m^2^ h) and the water content in the permeate was consistently above 99.8 wt.% during the investigated period. The excellent water-selectivity of CHA zeolite membranes, together with their remarkable stability, suggests that it has great potential for alcohol dehydration.

**Figure 8:**
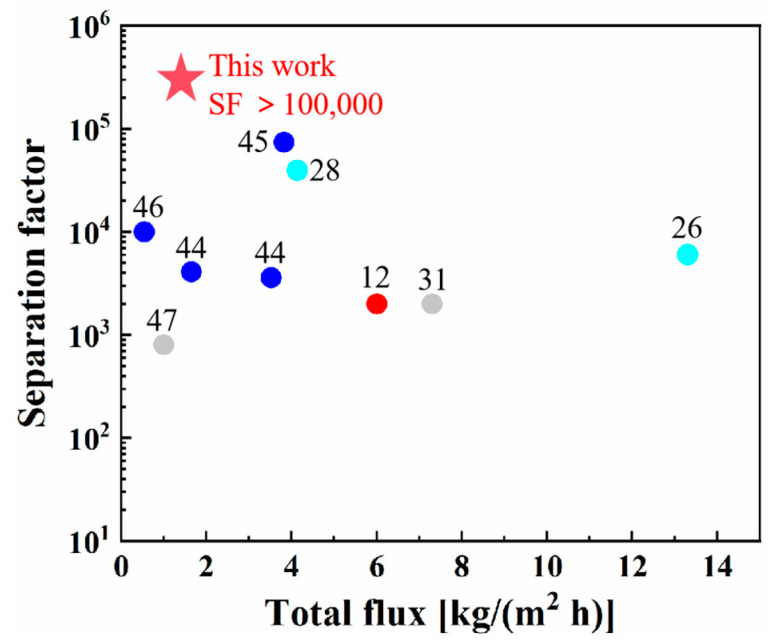
Comparison of PV performance of our membrane M6 with the reported ones in a 10/90 wt.% H_2_O/EtOH mixture at 348–353 K.

Figure 8 and Table 4 show the comparison of PV performance between our membranes and other dehydrated membranes in a 10/90 wt.% H_2_O/EtOH mixture. Most reported zeolite membranes for EtOH dehydration were NaA and CHA zeolite membranes with a Si/Al ratio of less than 3.5. NaA zeolite membranes (Si/Al ratio = 1) displayed a total flux of 1.65 kg/(m^2^ h) and a water/EtOH separation factor of 4100 [33]. In this work, template-free-synthesized CHA zeolite membranes with an increased Si/Al ratio (Si/Al = 5.3) had a separation factor higher than 100,000 for 10/90 wt.% H_2_O/EtOH, which was the highest one among the reported membranes in Figure 8 and Table 4. Although our membrane had a higher Si/Al ratio, its low defect density could be the reason for the excellent selectivity for water/ethanol separation. The separation performance of the membrane was comparable to the reported ones, as shown in Figure 8 and Table 4. It indicates that our high-quality CHA zeolite membrane has potential applications for ethanol dehydration, especially for the purification of ethanol with ultra-high purity requirements, such as the purification of electronic grade anhydrous ethanol for the semiconductor industry.

## 4. Conclusions

A novel CHA zeolite membrane with an increased Si/Al ratio of 5.3 was synthesized using an organic template-free method via the epitaxial growth of high-silica SSZ-13 seeds. The use of cesium and sodium mixed cations is crucial for the formation of a continuous and dense CHA zeolite membrane. Membrane synthesis displayed good reproducibility. The flux of the membrane rose with increasing temperature and feed water content. The typical CHA zeolite membrane exhibited a water/EtOH separation factor higher than 100,000, together with a total flux of 1.1 kg/(m^2^ h) at 348 K in a 10/90 wt.% H_2_O/EtOH mixture. The membrane also displayed excellent separation performance in water/isopropyl alcohol and water/methanol mixtures. It indicates the current membranes with enhanced Si/Al ratios have the potential for stable separation of water/alcohol mixtures.

## Figures and Tables

**Table 1:** EDX results from Figure 5 for a CHA zeolite membrane prepared under optimized conditions and SSZ-13 seeds.

	Si (Atomic%)	Al K (Atomic%)	Si/Al Ratio
M3	84.24	15.76	5.3
SSZ-13 seeds	93.41	6.59	14.2

**Table 2:** Pervaporation performance of membranes fabricated for different times (10/90 wt.% H_2_O/EtOH, 348 K).

Sample	Synthesis Time (h)	Permeate (H_2_O wt.%)	Flux [kg/(m^2^ h)]	Separation Factor
M1	24	99.79	2.0	4300
M2	36	99.82	1.6	6600
M3	48	99.94	1.1	16,600
M4	48	99.98	1.4	31,600
M5	48	>99.99	1.1	>100,000
M6	48	>99.99	1.2	>100,000
M7	72	99.13	1.0	1000
M8	72	99.25	0.6	1300

**Table 3:** VP performance at 378 K of membrane M2 in MeOH, EtOH, and IPA aqueous mixtures.

Feed	Perm (H_2_O wt.%)	Flux [kg/(m^2^ h)]	Separation Factor
10/90 wt.% H_2_O/EtOH	99.7781	1.8	4000
10/90 wt.% H_2_O/IPA	99.3903	2.5	1500
10/90 wt.% H_2_O/MeOH	97.7295	0.6	270
30/70 wt.% H_2_O/MeOH	98.8528	1.7	510

**Table 4:** PV performance of our membrane M5 and reported ones in a 10/90 wt.% H_2_O/EtOH mixture at 348–353 K.

Membranes	Flux [kg/(m^2^ h)]	Separation Factor	References
Cu-LTA	3.52	3600	[44]
Na-LTA	1.65	4100	[44]
NaA	3.82	73,800	[45]
NaA	0.54	>10,000	[46]
Silica	1	800	[47]
CHA	4.14	39,500	[28]
CHA	13.3	6000	[26]
CHA	7.3	2000	[31]
CHA	6	2000	[12]
CHA	1.2	>100,000	This work

## Data Availability

The original contributions presented in the study are included in the article, further inquiries can be directed to the corresponding author.
